# Investigating the orthotic effect of a passive gravity-compensated exoskeletal device on upper-limb function in people with multiple sclerosis: a pilot cross-sectional study

**DOI:** 10.1186/s12984-025-01715-8

**Published:** 2025-08-26

**Authors:** Thomas Bowman, Alessandro Torchio, Ilaria Carpinella, Tito Dinon, Erica Grange, Rachele Di Giovanni, Claudio Marcello Solaro, Davide Cattaneo, Marco Caimmi

**Affiliations:** 1 IRCCS Fondazione Don Carlo Gnocchi, Milan, Italy; 2 Institute of Intelligent Industrial Technologies and Systems for Advanced Manufacturing, National Research Council of Italy, Milan, Italy; 3 Scientific Research Area, Italian Multiple Sclerosis Foundation, Via Operai 40, Genoa, 16149 Italy; 4 Department of Rehabilitation, CRRF “Mons. Luigi Novarese”, Moncrivello, VC Italy; 5 Department of Neurology, Galliera Hospital, Genova, Italy; 6 Department of Pathophysiology and Transplantation, University of Milan, Milan, 20100 Italy

**Keywords:** Rehabilitation, Upper limb, Multiple sclerosis, Exoskeleton, Assistive device

## Abstract

**Background:**

Multiple Sclerosis (MS) is a neurodegenerative disorder causing lower and upper-limb (UL) impairments and significantly affecting independence. Current assistive technologies for UL rehabilitation in People with MS (PwMS) rely on actuated robotic systems, which present high costs and complexity. Passive gravity-compensated exoskeletons represent a promising alternative; however, their functional benefits remain underexplored. This study aimed to redesign and evaluate the orthotic effect of a passive gravity-compensated exoskeleton device in supporting upper-limb function in PwMS.

**Methods:**

This pilot cross-sectional study presents two phases: (I) redesigning an existing passive exoskeleton to improve usability and adaptability; (II) evaluating the orthotic effect and usability of the redesigned exoskeleton in a cohort of PwMS. Functional performance was assessed using the Action Research Arm Test (ARAT) and a modified Box and Block Test (mBBT) under *Exoskeleton Supported* and *Exoskeleton Unsupported* conditions. Kinematic parameters were extracted from three instrumented ARAT items, and usability was assessed with the System Usability Scale (SUS).

**Results:**

Phase I led to an iterative refinement of the exoskeleton, incorporating feedback from three PwMS and two therapists. In Phase II, thirteen PwMS (age: 59 [55–69] years; 10 males; EDSS: 7.5 [6.5-8.0] points) with different UL disabilities were recruited. Four participants with severe impairments increased the UL movement (orthotic effect) by 35.8% [29.0%–41.9%] and 24.1% [14.9%-33.3%] in the vertical and anteroposterior direction, with 8 [6.5–9.25] points improvements in the overall ARAT. Conversely, three individuals with mild UL disability needed 2.63 [2.17–3.45] seconds more to complete the instrumented ARAT items increasing the Jerk Index by 0.53[0.51–0.68]. The overall ARAT decreased by 7 [6–8] points and they transported 10[9–18] blocks less in the mBBT. The remaining participants with an FSS > 5.5 points, transported 9.5 [8-11.25] blocks more in the mBBT. The median SUS score was 70[62.5–70].

**Conclusions:**

Collaboration between therapists and engineers was key in refining the exoskeleton during phase I. Phase II results supported its positive orthotic effect for PwMS, particularly for those with moderate to severe UL impairments and fatigue. However, for individuals with mild deficits, the device may alter movement dynamics, affecting execution quality. Future improvements should focus on reducing bulk for clinical use. Additionally, studies on larger populations are needed to validate these findings.

**Supplementary Information:**

The online version contains supplementary material available at 10.1186/s12984-025-01715-8.

## Introduction

Multiple Sclerosis (MS) is a complex neurodegenerative disorder characterized by various symptoms, including motor, sensory, and cognitive deficits. Among these, upper-limb impairment holds particular significance, as it significantly impacts the daily functioning and independence of People with MS (PwMS). Studies by Bertoni and Solaro have provided essential insights into the prevalence, progression, and novel interventions for upper-limb dysfunction in MS, emphasizing the need for targeted and adaptable rehabilitation strategies [1–3]. Technological advancements in neurorehabilitation have increasingly focused on assistive devices, particularly robotic and electromechanical systems, to address the challenges associated with upper-limb impairments [4–6]. These devices have demonstrated potential in enhancing PwMS’s functional capabilities and quality of life [7]. However, most of the research and reviews, including the comprehensive scoping review by Gandolfi et al. [8], have primarily investigated actuated systems with powered components. While these technologies are valuable, they often come with limitations such as high costs [9].

In contrast, passive exoskeletal solutions, which lack active actuation and rely on mechanical elements like springs or elastic components, offer distinct advantages. These include reduced weight, lower cost, minimal maintenance, and usability in environments without power access. A recent work by Preethichandra et al. highlights the potential of passive exoskeletons across various domains, including rehabilitation [9]. Their analysis underscores how these systems provide effective gravity compensation, energy recycling, and user-friendly designs, making them particularly suitable for addressing upper-limb impairments in populations like PwMS. Passive exoskeletons suffer from a lack of precise motion control compared to actuated exoskeletons with control algorithms, but in the context of MS rehabilitation the focus may be more on functional support and endurance improvement than on precision control.

Despite these advantages, passive exoskeletons remain underexplored in the context of neurorehabilitation for MS. Most existing designs focus on industrial applications, and there is a notable lack of studies addressing their usability, clinical effectiveness, and specific design considerations tailored to PwMS [9–12]. This gap is significant, given the unique challenges faced by PwMS, such as muscle weakness and fatigue which demand lightweight and ergonomically optimized solutions.

This paper seeks to bridge the aforementioned gap by investigating the “orthotic effect” of a passive gravity-compensated exoskeletal device designed to assist upper-limb function in PwMS. This effect refers to the immediate impact of using the passive gravity-compensated exoskeleton on motor performances compared to the same task performed without the exoskeleton. By incorporating insights from both clinical needs and engineering advancements, this study aims to contribute to the development of cost effective and clinically viable assistive technologies.

Specifically, the first aim (Phase I) of the study was to redesign the exoskeleton, developed by STIIMA and originally known as “LIGHTarm” [13, 14], to meet the specific needs of PwMS. This includes leveraging its unique kinematics and back-drivability to provide effective and comfortable upper-limb support during rehabilitation.

The second aim (Phase II) was to verify the “orthotic effect” of the redesigned passive exoskeleton, focusing on its ability to assist motor performance in PwMS.

Finally, we addressed usability concerns associated with the use of the device in this population to propose practical design principles for further optimization.

## Materials and methods

In alignment with the study aims, this section describes the methodology across two distinct phases. Phase I focuses on the redesign of the LIGHTarm exoskeleton, incorporating feedback from volunteers with MS and therapists (physical and occupational therapist) who tested the original device. The original LIGHTarm exoskeleton supports complex functional movements, thanks to its kinematics, which align closely with natural shoulder and elbow motion. Notably, the hand remains unconstrained, allowing for object manipulation during task execution. A comprehensive technical description of LIGHTarm can be found in the studies by Scano et al. and Spagnuolo et al. [13, 14]. Critiques provided by users informed targeted improvements in its usability, comfort, and functionality. The outcome of this iterative development process is the redesigned exoskeleton, renamed ASSISTarmMS, which addresses the limitations identified. Phase II encompasses a cross-sectional study to evaluate the orthotic effect and usability of ASSISTarmMS. This phase involves a pilot group of PwMS, assessing their functional capabilities and user experience while employing the redesigned exoskeleton.

### Phase I: redesign of the LIGHTarm exoskeleton

**Fig. 1:**
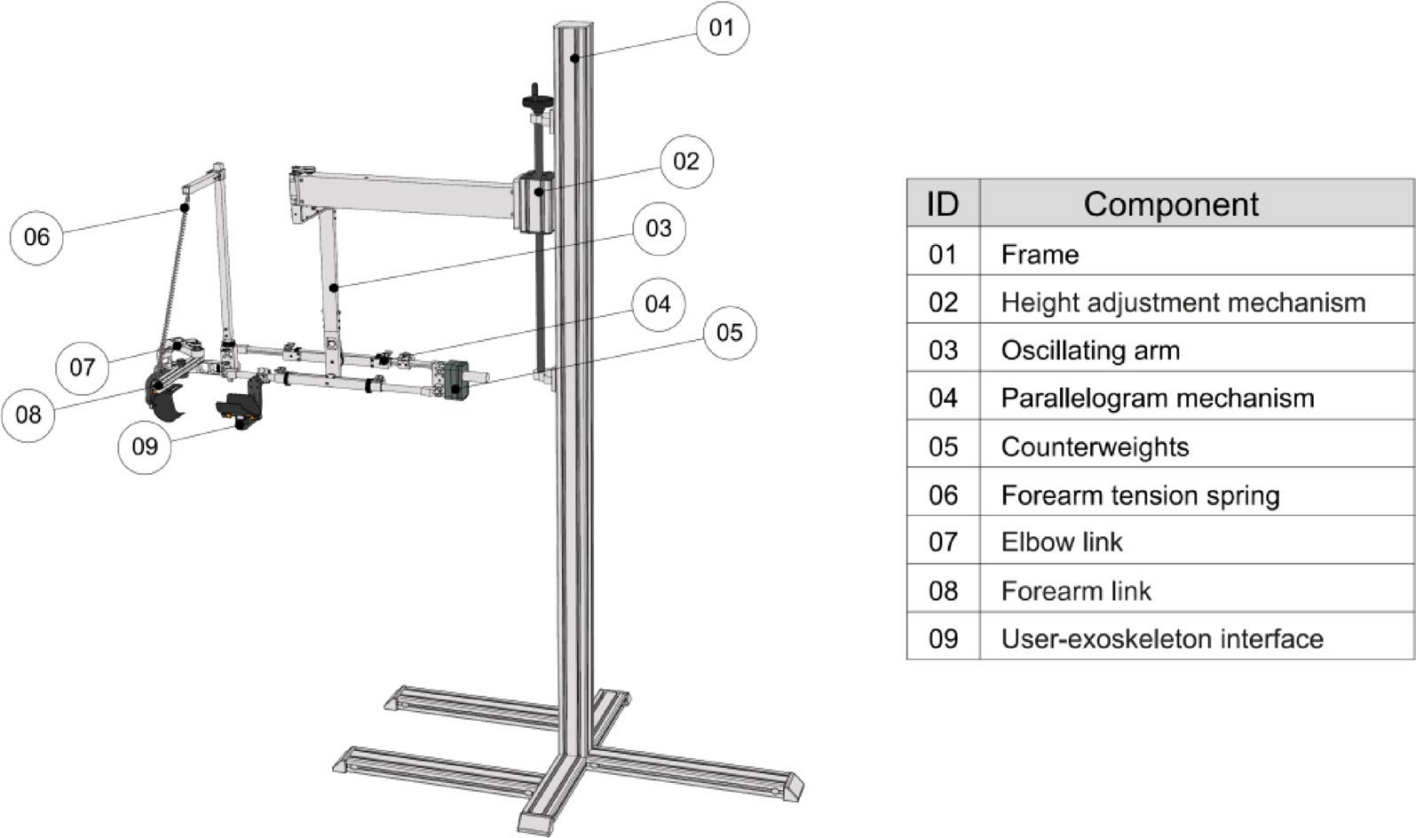
LIGHTarm exoskeleton used during the ergonomic assessment. The main functional components of the system are numbered in the image and listed in the table on the right side of the figure

Three PwMS (two females and one male; ages 51–67 years) with a wide range of disability (EDSS scores: 2.5–7.5) participated in a preliminary ergonomic assessment of the LIGHTarm, facilitated by two trained therapists. The goal was to identify potential usability issues and adapt the exoskeleton to better meet the needs of both PwMS and therapists. The MS participants involved in this ergonomic evaluation, referred to as Participant 1, Participant 2, and Participant 3, also took part in the cross-sectional study described in Phase II. The version of LIGHTarm used during this assessment is shown in figure 1, which also highlights its main functional components.

#### Redesigning

The key aspects identified after the ergonomic assessment were:

1. Maximizing safety for PwMS.
2. Facilitating donning operations for clinical therapists.
3. Improving comfort for PwMS.
4. Enabling mechanical adjustments for clinical therapists.

**Fig. 2:**
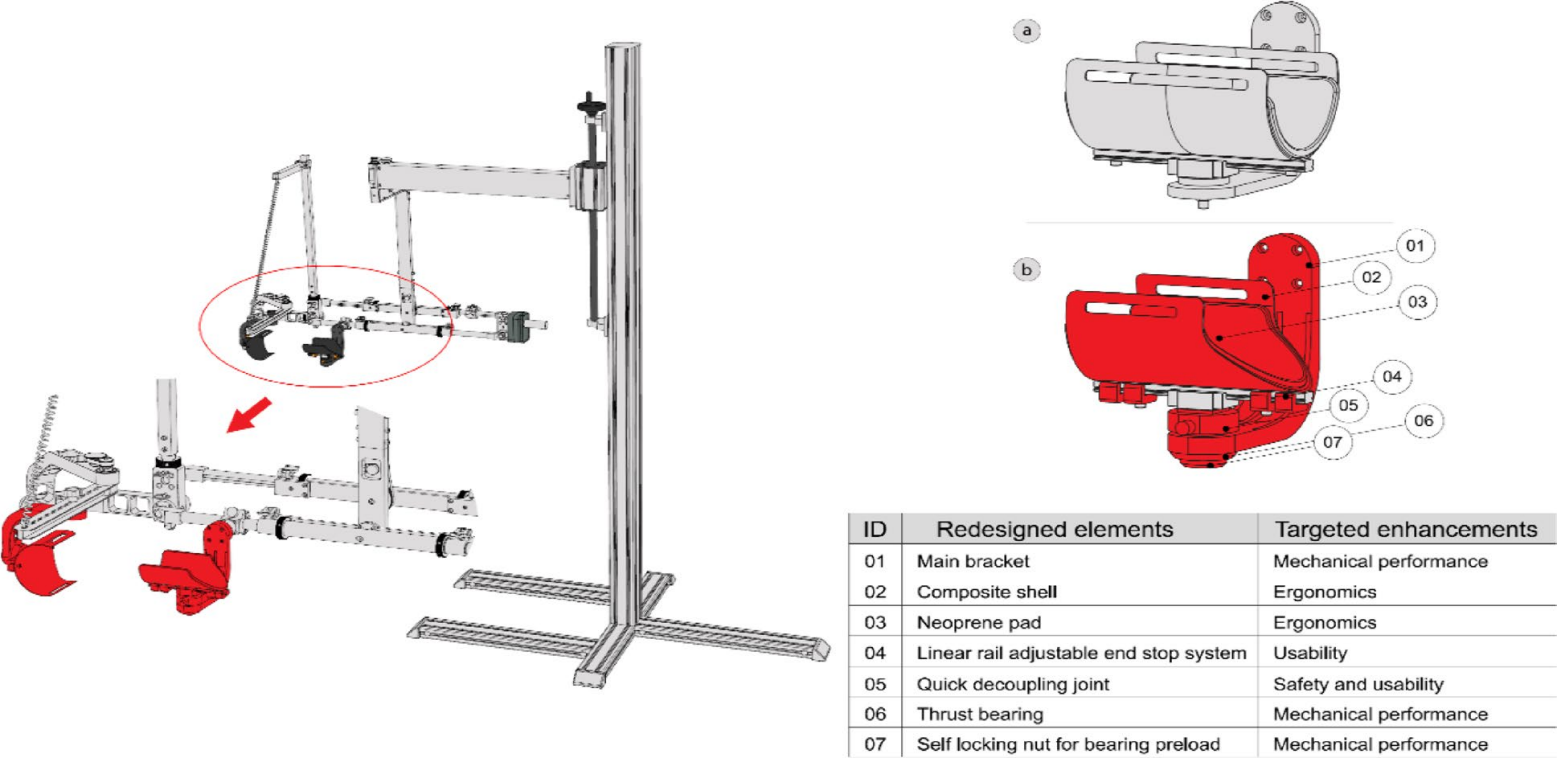
Redesign of the subassemblies connecting the user to the exoskeleton (left panel, red parts). The right panel shows the LIGHTarm user-device interface: (a) upper image, original configuration; (b) lower image, redesigned components highlighted in red

The areas of intervention focused on the interfaces between the user and the device, specifically through the redesign of two subassemblies connecting the user to the exoskeleton: one for the forearm and one for the arm (Fig. 2, left panel, red parts). The right panel of figure 2 highlights the redesigned components intended to address the identified needs.

**Fig. 3:**
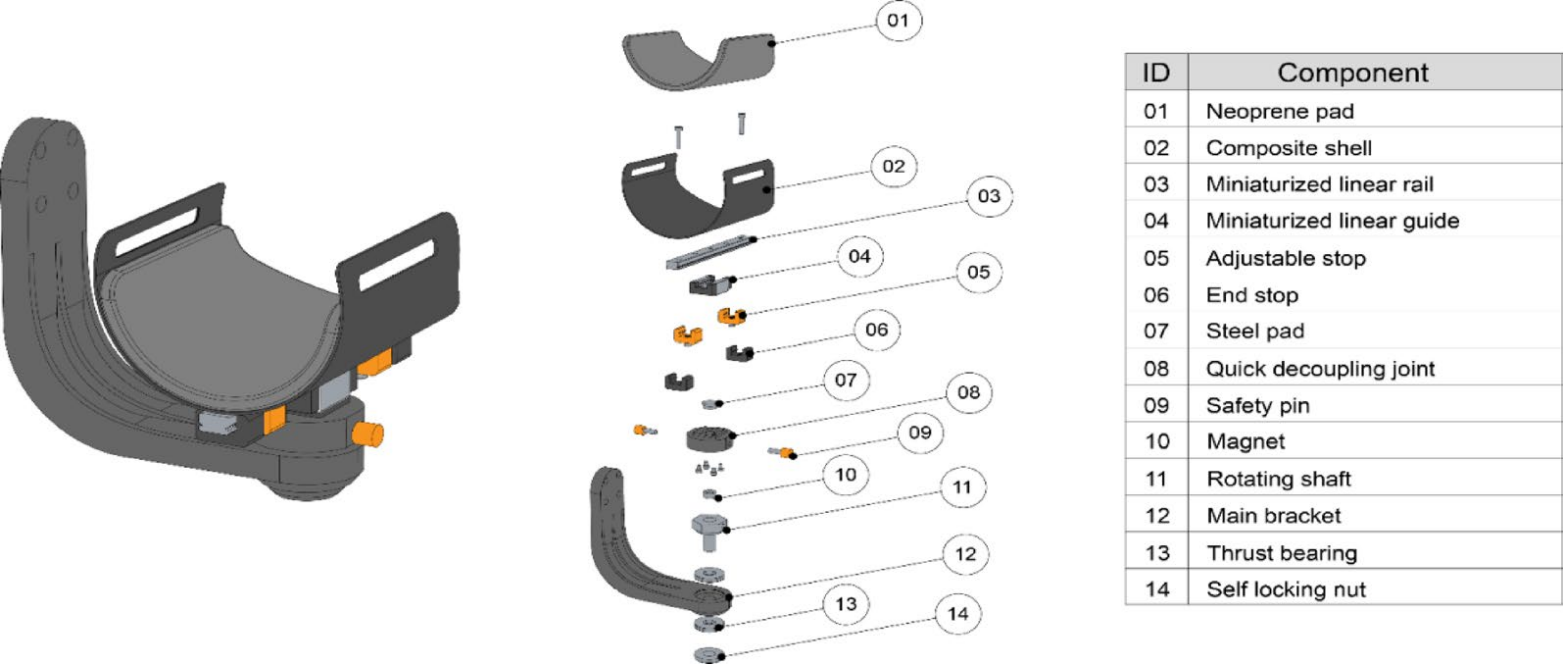
Subassembly specifications and components. The orange items are the ones which could be operated by the PT to perform the donning/doffing operations or to change the travel of the linear rail the device to different users

These two subassemblies are very similar in design and share the same operating principle. The user’s arm is secured to a carbon-fiber shell (Fig. 3, ID 02) using a velcro strap. This shell can slide back and forth via a miniaturized linear rail (Fig. 3, ID 03/04). The rail is connected to a rotating steel shaft (Fig. 3, ID 11) through a custom quick-decoupling joint (Fig. 3, ID 08) and can rotate due to a pair of adjustable thrust bearings (Fig. 3, ID 13). These bearings are incorporated into the main additively manufactured PC-ABS bracket (Fig. 3, ID 12), which is responsible for attaching the entire subassembly to the main body of the exoskeleton. The balancing system to support the user’s arm, made of counterweights (Fig. 1- ID 05), was not modified.

##### User safety

**Fig. 4:**
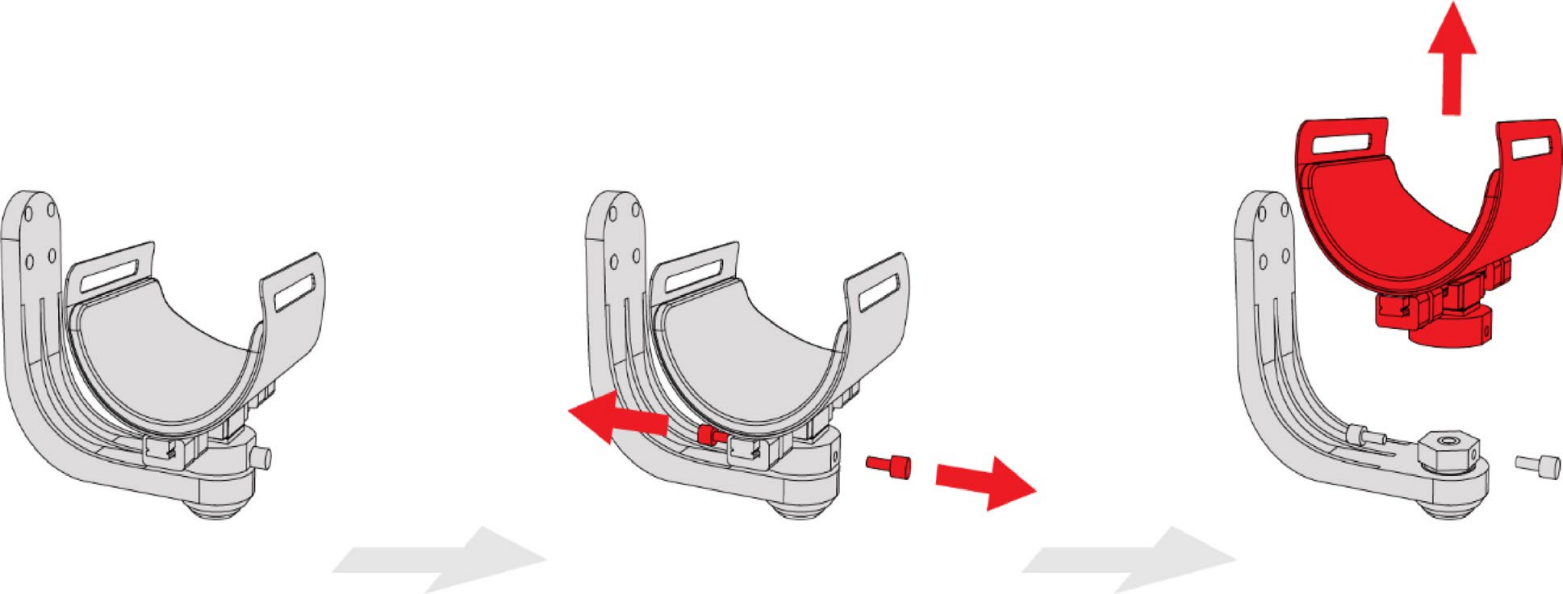
The decoupling mechanism of the subassembly

In the original LIGHTarm design it was not possible to quickly disconnect the user, as the subassemblies were rigidly connected to the device’s structure, and the user’s arm and forearm were secured with velcro straps. This posed a potential safety risk, particularly in emergencies. To address this, a custom decoupling joint (Fig. 3, ID 08) was developed, allowing for mechanical separation between the linear rail and the main bracket (Fig. 4).

ASSISTArmMS features two such decoupling joints, one for each connection between the exoskeleton and the user. Each joint can be manually operated by the therapists in case of emergency, enabling rapid disconnection from the device. To detach the shell from the exoskeleton, two aluminium safety pins (Fig. 3, ID 09) must be removed from the subassembly. These pins are permanently bonded to neodymium magnets with structural adhesive, ensuring a secure connection during regular use. However, in an emergency, the magnets allow for quick and easy removal. Once both pins are removed, the shell can be effortlessly pulled away from the device. This modification significantly speeds up the doffing process in emergencies, particularly when dealing with non-compliant users (e.g., in cases of sudden illness).

##### Donning and doffing

Phase I testing revealed that the donning and doffing operations were overly complex and time-consuming, rendering them impractical for clinical trials and rehabilitation sessions. These inefficiencies highlighted the need for improvement, particularly in streamlining the process to ensure compatibility with the demands of a clinical environment. As a result, significant efforts were made to enhance the design and simplify these procedures. The decoupling operation described in the previous section can be performed in the same manner whether the user is connected to the shell.

**Fig. 5:**
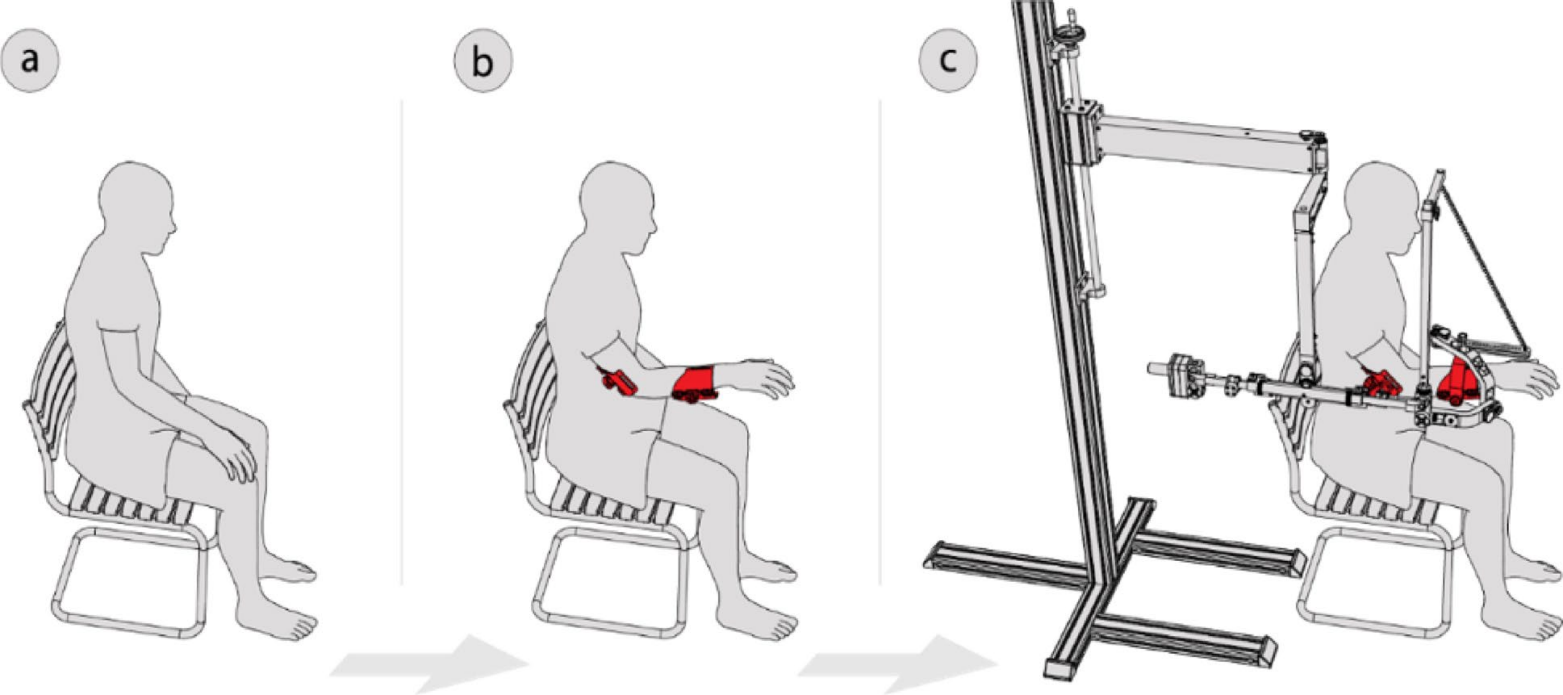
Donning procedures: (a) the user is sitting waiting for the shells; (b) both shells are positioned; (c) the user is linked to ASSISTArmMS

This feature can be leveraged to facilitate the donning operation by inverting the sequence of operations described above. With this redesign, it is possible to comfortably position the user and attach both shells before linking the arm and forearm to the device (Fig. 5). This is done by aligning the two halves of the decoupling joint and securing them with the safety pins.

Each decoupling joint is equipped with a male-to-female hexagonal connection, ensuring easy and accurate alignment of the safety pins within their slots. Additionally, a magnet provides a temporary connection between the two halves of the joint during the donning operations. The male part of the joint is fabricated from steel using turning and milling processes, while the female part is produced through additive manufacturing with PC-ABS polymer. The magnet is permanently bonded inside the component using structural adhesive. By eliminating the need to dress the users while simultaneously interacting with the mechanical structure of the device, this procedure reduces the overall time required for donning and doffing the exoskeleton at the beginning and end of each session. This new approach significantly decreases stress and discomfort of the user while enhancing ergonomics for the therapists performing the dressing and adjustment operations.

##### User comfort

In the original LIGHTarm design, only a single shell size was available, designed to fit the arms of healthy individuals. However, PwMS often have thinner arms due to muscle weakness caused by the disease, leading to discomfort and slipping between the arm and the shell. This misalignment compromised the system kinematics, making it a critical issue to address in the redesign.

ASSISTarmMS now features three interchangeable rigid shells (Fig. 3, ID 02) in sizes small (S), medium (M), and large (L). A neoprene pad (Fig. 3, ID 01) is positioned between the shell and the user’s skin, which can be removed for cleaning and swapped with pads of varying thickness to accommodate a wide range of arm sizes. Additionally, user comfort can be further enhanced with an adjustable velcro strap.

##### Mechanical adjustments

Phase I testing revealed that adjusting the exoskeleton to accommodate different anthropometric measurements often resulted in inadequate travel ranges, which could be either too long or too short. This led to jerky movements and misalignments, particularly between the elbow-joint center of rotation and the corresponding exoskeleton joint. These misalignments compromised the smoothness and accuracy of the movement, highlighting the need for adjustable travel ranges to ensure proper alignment and functionality for PwMS.

In the redesigned version of the links, a new set of additively manufactured PC-ABS mechanical stops has been introduced, allowing for user-specific adjustments to the shells travel range along the linear rails. To further minimize unwanted mechanical play between the rotating elements, adjustable thrust bearings and a specially designed fine-pitched, self-locking threaded nut were incorporated.

#### Phase I—key findings

The evaluation of the LIGHTarm exoskeleton identified significant limitations, including safety risks from the lack of quick-release mechanisms, user discomfort due to rigid shell sizes, lengthy and complex donning and doffing procedures, and insufficient adjustability for varying anthropometric needs. The redesign into the ASSISTarmMS addressed these issues by incorporating a quick-disconnect mechanism for enhanced safety, customizable shells and padding for greater comfort, and simplified donning and doffing processes to improve usability. Adjustable mechanical components allowed better tailoring to individual users’ needs, enhancing adaptability. After these improvements the system should be more user-friendly, comfortable, and safe, while retaining the beneficial kinematics of the original design. Importantly, the enhancements rendered the ASSISTarmMS suitable for Phase II trials, facilitating the cross-sectional study to evaluate its orthotic effects and usability. The work also highlighted critical design principles for assistive devices, emphasizing the importance of balancing technical performance with user-centered factors such as comfort, fit, and ease of use to optimize functionality in real-world applications. Table 1 summarizes the issues identified in LIGHTarm, the associated limitations, and the solutions implemented.

**Table 1:** Summary of LIGHTarm design issues, related limitations, and implemented improvements

Issue	Limitation	Solution
No possibility to quickly disconnect the user from the device	Safety	Implementation of a custom decoupling joint
Overly complex and time-consuming donning/doffing operations	Safety, therapist/user ergonomics, comfort, time	Implementation of a custom decoupling joint
Single shell size	User comfort	Design of multiple shell sizes
Arm slipping inside the rigid shell	User comfort, mechanical performance reduction	Use of interchangeable and removable neoprene pads
Arm slipping inside the rigid shell	User comfort, mechanical performance reduction	Use of Velcro straps
Inadequate travel ranges	Mechanical performance reduction	Introduction of adjustable end stops
No possibility to regulate bearing preload (system transparency)	Mechanical performance reduction	Introduction of adjustable thrust bearings and fine-pitched, self-locking nut

### Phase II: cross-sectional experimental study

Throughout phase II of this study, the exoskeletal device ASSISTarmMS was employed in a cross-sectional study design to test its orthotic effect in a pilot group of PwMS.

#### Participants

PwMS were recruited from the MS rehabilitation department of the Fondazione Don Gnocchi (Milan, Italy) between July 2020 and January 2022. Inclusion criteria were: diagnosis of MS confirmed by a neurologist, age > 18, elbow extension Manual Muscle Test (MMT) ≥ 2 (active movement without gravity), shoulder flexion MMT ≤ 4 (active movement against resistance), 9 Hole Peg Test >25 s and capability to perform gross hand movement such as “bring the hand to the mouth or the head”, and the ability to follow the protocol and provide informed consent. Participants were excluded if they had contraindications to using the exoskeleton (e.g., severe joint ROM limitations, fractures, severe osteoporosis); cardiac pacemakers, defibrillators or other electronic implants; or were taking medications that could potentially affect cerebellar function or muscle tone. These medications included anti-epileptic drugs, benzodiazepines, antidepressants, beta-blockers, and spasticity drugs initiated within 2 weeks prior to recruitment. Additional exclusion criteria included the coexistence of major other neurological, neuropsychological, or psychiatric diseases, as confirmed by clinical history.

#### Experimental procedures

All participants underwent clinical and instrumented assessments, starting with the evaluation of the outcome measures to characterize the sample clinically. The orthotic effect assessment was then conducted under two experimental conditions: *Exoskeleton Supported* and *Exoskeleton Unsupported*. Each participant’s most clinically compromised upper limb was evaluated under both conditions, adhering to the study inclusion and exclusion criteria. During the *Exoskeleton Supported* condition the ASSISTarmMS was set up according to the user’s arm weight and anthropometric measurements to ensure the most comfortable and efficient configuration. Specifically, the therapist adjusted the mechanical components of the device according to the specific user’s anthropometric needs and calibrated the level of assistance by compensating the arm weight. This was achieved by fine-tuning the device balancing counterweight (Fig. 1) according to the user’s preference [13]. Calibration involved two to three forward flexion movements, starting with the hand resting on the table and progressing to the anterosuperior limit required by the functional task. Based on the range of motion achieved and the user’s subjective feedback, weights were either added or removed to appropriately unload the upper limb. The participant was then asked to perform maximal horizontal adduction and abduction movements, and any reported restrictions or discomfort were noted. Additionally, the height of the seat, the position of the table and the distance from the assessment instruments were adjusted to ensure that participants began the assessments from the same starting position. The time required for donning and calibrating the device depended on the participant’s level of compliance. However, once seated on the standardized chair used for all participants, the average time was approximately 5 min. The order of the experimental conditions was randomized for each participant to minimize bias. At the end of the experimental procedures, each participant assessed the usability of the device. All assessments were performed by two trained therapists.

##### Clinical assessment

The following clinical tests were conducted to characterize the participants from a clinical and functional point of view before assessing the orthotic effect of the ASSISTarmMS.

- *Expanded disability status scale (EDSS)*: is a method of quantifying disability in PwMS and monitoring changes in the level of disability over time. The scale ranges from 0 to 10, with 0 indicating normal neurological function and 10 indicating death due to MS [15].
- *Medical Research Council Manual Muscle Testing Scale (MMT)*: is a widely used tool to assess the strength of individual muscles or muscle groups. The MRC scale grades muscle strength on a scale from 0 to 5, based on the subject’s ability to contract a muscle against resistance [16].
- *Modified Ashworth Scale (MAS)*: measures spasticity in subjects with lesions to the central nervous system. MAS is an assessment used to measure the increase in muscle tone. MAS assigns an ordinal grade of spasticity from 0 (no spasticity) to 4 (severe spasticity) [17].
- *Nine Hole Peg Test (9HPT)*: is a standardized, quantitative assessment used to measure finger dexterity [18]. The test is administered by asking the client to take the pegs from a container, one by one, and place them into holes on the board as quickly as possible. Participants must then remove the pegs from the holes, one by one, and put them back into the container. Scores are based on the time taken to complete the activity and are recorded in seconds.
- *Box and Block Test (BBT)*: is a test to assess unilateral gross manual dexterity [19]. The individual is placed in front of a box with two compartments and is instructed to move as many blocks as possible, one at a time, from one compartment to the other for 60 seconds.
- *Fatigue Severity Scale (FSS)*: is a 9-item questionnaire with questions related to how fatigue interferes with certain activities and rates its severity according to a self-report scale [20]. The items are scored on a 7-point scale with 1 = strongly disagree and 7 = strongly agree.

##### Orthotic effect assessment

The Action Research Arm Test and a modified version of the BBT were used as primary outcomes of the study. Both tests were conducted in *Exoskeleton Unsupported* and *Exoskeleton Supported* conditions.

##### Assessment procedure

**Fig. 6:**
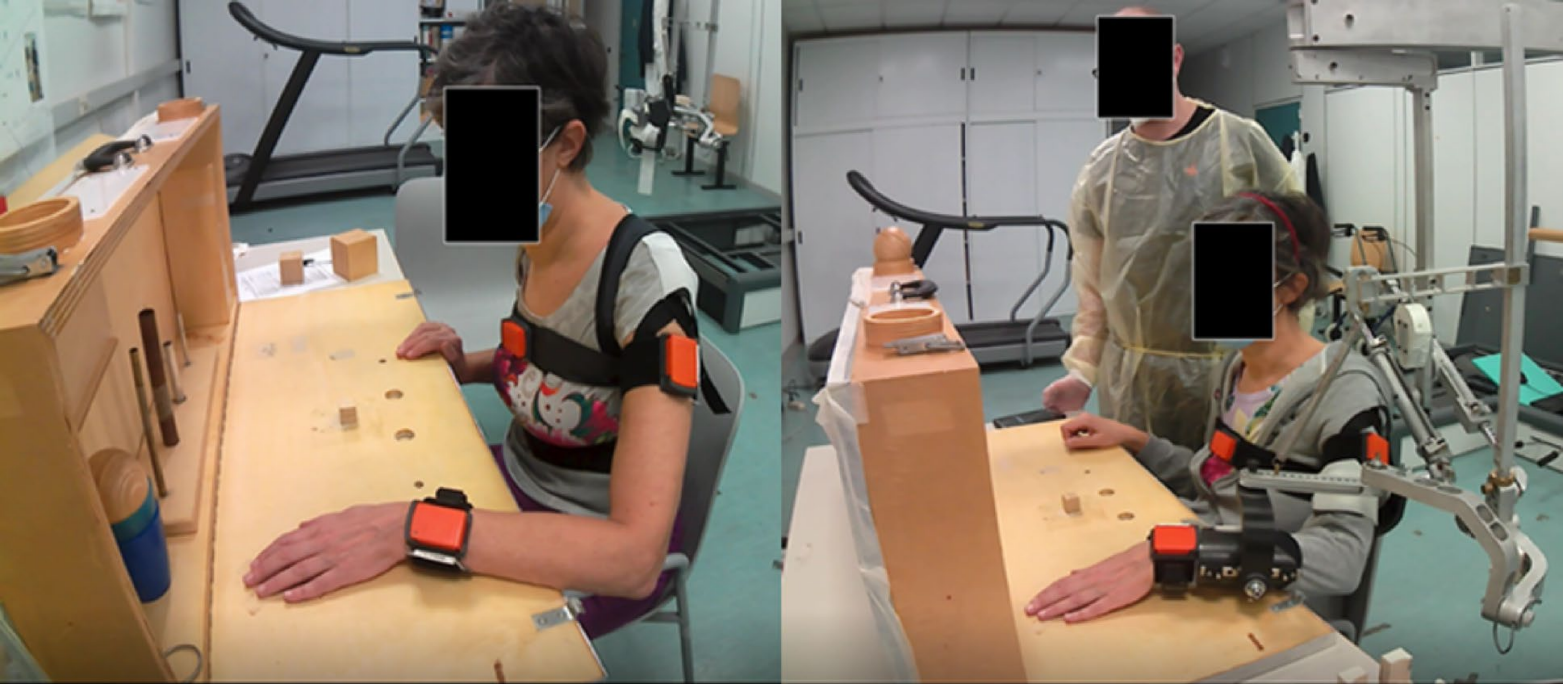
Set up for the assessment of the three instrumented ARAT items: Exoskeleton Unsupported (left) and Exoskeleton supported (right)

*Action Research Arm Test (ARAT)*: This is a 19-item measure divided into 4 sub-tests (grasp, grip, pinch, and gross arm movement) to assess upper-limb functioning. Each item is timed and scored (0–3 points, 3 = best score) on the success of the execution and the quality of the movement [21]. All 19 items were clinically assessed in both the *Exoskeleton Unsupported* and *Exoskeleton Supported* conditions (Fig. 6), while, to simplify the evaluation procedure, only three representative items of the ARAT have been instrumented [22]. The instrumented items were: grasp a wood block with i) a 2.5 cm edge and ii) a 5 cm edge, iii), pinch a ball bearing with 2nd finger and thumb. In these items, the instructions were to move these objects from the table and place them on a shelf placed at shoulder height and at a distance equal to the sum of the lengths of the arms and forearm starting with the hand on the table. These items were chosen as they imply both grasp and pinch grip requiring to move the objects not only in the horizontal plane but also vertically against gravity, making them particularly suitable for use with the exoskeleton. The three instrumented items of the ARAT were recorded with a video camera in both experimental conditions.

The instrumentation consisted of three inertial measurement units – IMU (MTw, XSens, The Netherlands) fixed through elastic bands to the chest at the sternum level, to the lateral side of the upper arm at the proximal third of the segment between the elbow and shoulder, and to the dorsal side of the forearm at wrist level (Fig. 6). Each IMU is made up of a three-dimensional accelerometer (±160 m/s^2 range), a three-dimensional gyroscope, (±1200 deg/s range), and a three-dimensional magnetometer (±1.5 Gauss range).

*Modified Box and Block Test (mBBT)*: We conducted a modified version of the original test to assess upper-limb endurance during gross manual activities. To be consistent with the existing literature [23], we extended the test duration, instructing participants to move as many blocks as possible, one at a time, from one compartment of the box to the other, within a 180-second time frame (i.e., the original version of the test lasts 60 seconds).

##### Usability assessment

Participants completed the System Usability Scale (SUS) questionnaire at the end of the assessment procedure. The SUS is a simple, ten-item questionnaire used to evaluate the usability of a device, system, or service. It provides a quick, reliable way to measure user satisfaction and perceived usability. Participants rated their agreement on a 5-point Likert scale (from “Strongly Disagree” to “Strongly Agree”). Usability is considered to be “*poor”* for SUS score between 0 and 50, “*ok”* for SUS score between 50 and 74, and “*good”* for SUS score > 70. A score of 68 is considered as a threshold score for acceptability and 80.3 is the threshold for a full positive evaluation. The maximum score is 100 [24].

#### Data analysis and statistics

Data from the three instrumented items of the ARAT were elaborated to calculate the kinematic outcome measures. The IMU orientation in space (i.e., the rotation matrix) was estimated from raw signals by a sensor fusion algorithm based on a Kalman Filter implemented on a Digital Signal Processor embedded in the sensor. In the case of high-functioning participants able to complete the three ARAT items without the support of the exoskeleton, only the wrist sensor was used to compute the normalized Jerk Index and the duration of each item, following Carpinella et al. [25]. When this procedure was not applicable because the participant was able to perform only a part of the items with and/or without the ASSISTarmMS, the procedures described by Caimmi et al. 2019 for estimating the position of the IMUs were applied [22]. The anteroposterior (AP) and vertical (VT) displacement of the wrist, from the starting position on the table to the final position reached by the participant during each ARAT item, was estimated following a three-link kinematic chain model based on the orientation of the three IMUs and scaled on each subject’s anthropometry [26]. To reduce errors due to a possible sub-optimal calibration of the IMUs and the IMUs-body segments alignment, the wrist displacement was expressed as a percentage of the nominal anteroposterior and vertical distance of the shelf from the initial position of the wrist. The reference values were obtained by therapists who passively performed the three ARAT items, displacing each participant’s hand while wearing the IMUs before the active execution of the participants. To ensure consistent spatial references, the therapists maintained the same IMU placement between experimental conditions, aiming not to remove the sensors from the participant’s arm during the donning procedures (Fig. 5). Median values of the kinematic parameters were calculated by combining the results from two of the three instrumented ARAT items (i.e., grasping a wooden block with a 2.5 cm edge and with a 5 cm edge) across the entire sample. The third item (pinching a ball bearing using the second finger and thumb) is presented separately in the Supplementary Materials (Supplementary Fig. 1), as it is clinically distinct from the other tasks and appears to require a higher degree of fine motor control. Regarding the usability assessment, we calculated the SUS score by adding up the converted responses and multiplying that total by 2.5 for each subject. The SUS final score was interpreted according to the literature [24]. The number of adverse events related to the use of the exoskeleton was recorded and categorized if they occurred.

Due to the small sample size non-parametric statistics was performed, medians and interquartile ranges [25th percentile – 75th percentile] were computed and the 2-sided Wilcoxon paired sign-test was used to verify the orthotic effect of the device on the whole group comparing the *Exoskeleton Unsupported* condition to the *Exoskeleton Supported* one. The individual orthotic effect has been calculated as the ARAT change score ($\:{\Delta\:}ARAT$= ARAT *Exoskeleton Supported* – ARAT *Exoskeleton Unsupported*) and the mBBT_change score ($\:{\Delta\:}mBBT$= mBBT *Exoskeleton Supported* – mBBT *Exoskeleton Unsupported*). Moreover, we calculated the Spearman correlation coefficient between the individual orthotic effect ($\:{\Delta\:}ARAT\:$and $\:{\Delta\:}mBBT$) and the MMT, MAS, ARAT *Exoskeleton Unsupported* and mBBT *Exoskeleton Unsupported* to investigate the association between participants’ clinical characteristics and the benefits or limitations they experience from using the device.

All analyses were performed using R software (version 4.1.0) and MATLAB R2017b (The MathWorks, Inc., Natick, MA, USA). Finally, the level of statistical significance was α = 0.05.

## Results

### Characteristics of the sample

Thirteen MS subjects completed the Phase II of the study. The sample included three females (23%) and ten males (77%) with a median [Q1-Q3] EDSS score of 7.5 [6.50-8.00] points, indicating a severe walking disability among most of the participants. Regarding hand dominance, during the experimental procedures only 6 out of 13 (46.2%) received exoskeleton assistance in their dominant arm. Table 2 reports detailed demographic information for each participant.

**Table 2:** Demographic characteristics of the sample

Subject n°	Age (years)	Gender	Disease duration (years)	EDSS	Dominant arm	FSS
1*	67	Female	29	7.5	R	6.22
2*	57	Female	13	6.5	R	6.44
3*	51	Male	11	2.5	L	2.78
4	59	Male	17	8	R	3.89
5	64	Male	46	8	R	5.89
6	54	Male	12	7.5	R	7.00
7	69	Male	23	8	R	7.00
8	55	Male	10	8	R	5.44
9	82	Male	20	7	R	3.89
10	75	Male	27	8.5	R	2.11
11	57	Female	42	6.5	R	6.78
12	53	Male	11	7	R	6.11
13	72	Male	25	4	R	4.00
Median [25th – 75th]	59 [55–69]	-	20 (12–27)	7.5 [6.5–8.0]	-	5.9 [3.9–6.4]
N (%)	-	Male: 10 (77%) Female: 3 (23%)	-	-	L: 1 (92%) R: 12 (8%)	-

*EDSS* Expandend Disabilty Status Scale,* FSS* Fatigue Severity Scale,* L* left,* R* right, [*25th – 75th]* 25th percentile − 75th percentile

* Participants who also took part in the ergonomic assessment of Phase I.

Participants exhibited varying levels of upper-limb disability, with a median of 13 [5–24] blocks on the BBT and 81.31 [61.86-120.35] seconds on the 9HPT in the assisted arm for those able to complete the tests. Table 3 reports detailed clinical characteristics of the investigated arm for each participant.

**Table 3:** Clinical characteristics of the supported arm

Subjects n°	Supported Arm	BBT (Blocks)	9HPT (s)	MMT Sh. Flex.	MMT Sh. Abd.	MMT Sh. IR.	MMT Sh. ER.	MMT Elb. Flex.	MMT Elb. Ext.	MAS Sh. Flex.	MAS Sh. Abd.	MAS Elb. Flex.	MAS Elb. Ext.
1 2 3 4 5 6 7 8 9 10 11 12 13	L L L L L R R R L R L R L	15 24 30 5 13 1 4 44 13 1 28 12 20	259.57 62.1 74.8 n.a. 87.82 n.a. n.a. 28.11 n.a. n.a. 61.15 159.5 107.3	3 3 2 2 2 2 2 4 2 3 3 3 3	3 3 2 2 2 2 2 4 2 3 3 3 3	4 3 3 3 4 3 3 4 4 4 4 4 4	3 3 2 2 3 2 2 4 2 3 3 4 3	3 4 3 3 5 3 3 5 5 4 3 4 4	3 3 3 3 5 2 3 5 4 3 4 5 4	1 0 0 0 0 1 2 0 1 2 0 0 0	1 0 0 0 1 1 2 0 1 2 0 0 0	0 1 0 0 1 1 1 0 0 2 0 0 0	0 0 0 0 1 1 2 0 0 3 0 0 0

*9HPT* Nine Hole Peg Test,* BBT* Box and Block Test,* MMT* Manual Muscle Test,* MAS* Modified Ashworth Scale,* Abd* Abduction,* Flex* Flexion,* Ext* Extension,* ER* External Rotation,* IR* Internal Rotation,* Sh* Shoulder,* Elb* Elbow,* n.a.* not able

### Orthotic effect

**Fig. 7:**
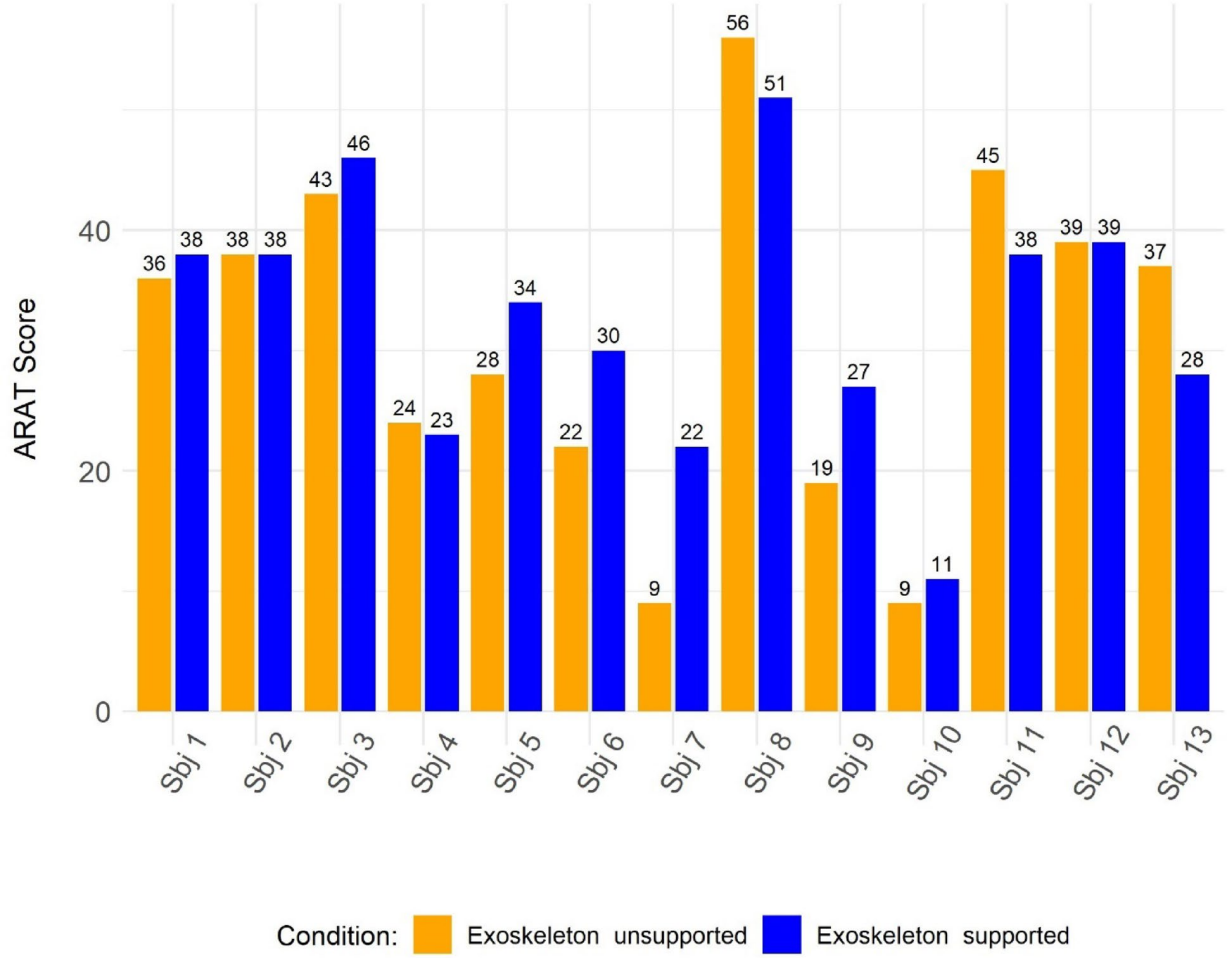
The orthotic effect of each participant performing the ARAT. Sbj: Subject; ARAT: Action Research Arm Test

The first part of the orthotic effect was evaluated based on both the overall ARAT results and the performance in the three instrumented ARAT items. Figure 7 shows the overall ARAT results of each participant. Overall, the group showed limited positive effect of the exoskeleton support, with a median change score between *Exoskeleton Supported* and *Exoskeleton Unsupported* conditions of $\:{\Delta\:}ARAT$ = 2 [-1– 6] points (W = 23, *p* = 0.37). However, seven participants (Sbj 1, Sbj 3, Sbj 5, Sbj 6, Sbj 7, Sbj 9, Sbj 10) achieved median higher scores in the *Exoskeleton Supported* condition (30 [24.5–36] points) compared to the *Exoskeleton Unsupported* condition (22 [14–32] points). With regard to the other six participants, four (Sbj 4, Sbj 8, Sbj 11, Sbj 13) had lower scores in the *Exoskeleton Supported* condition (33 [26.75–41.25] points) compared to the *Exoskeleton Unsupported* condition (41 [33.75–47.75] points), while Sbj 2 and Sbj 12 showed no change.

**Fig. 8:**
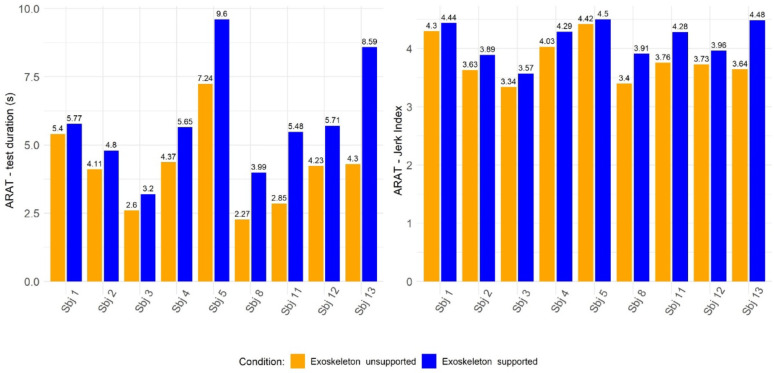
Median test duration (left) and median Jerk values (right) for each participant across the three instrumented ARAT tasks under the *Exoskeleton unsupported* and *Exoskeleton supported* conditions

Regarding the three instrumented items of the ARAT (*i.e., i)* grasping a wood block with a 2.5 cm edge, ii) grasping a wood block with a 5 cm edge, and iii) pinching a ball bearing with 2nd finger and thumb), nine out of thirteen (Sbj 1, Sbj 2, Sbj 3, Sbj 4, Sbj 5, Sbj 8, Sbj 11, Sbj 12 and Sbj 13) subjects were able to complete the three items in the *Exoskeleton Unsupported* condition. In this subset, the median time to perform the three instrumented items was 4.23 [2.85–4.37] seconds in the *Exoskeleton Unsupported* condition, compared to 5.65 [4.80–5.77] seconds in the *Exoskeleton Supported* condition (W = 0, *p* = 0.008). Regarding the Jerk Index, median values were 3.73 [3.63–4.03] in the *Exoskeleton Unsupported* condition and 4.28 [3.91–4.44] in the *Exoskeleton Supported* condition (W = 0, *p* = 0.008). The left panel of figure 8 reports the median time values for each participant in performing the three instrumented items of the ARAT test, while the right panel displays the corresponding median Jerk Index values.

**Fig. 9:**
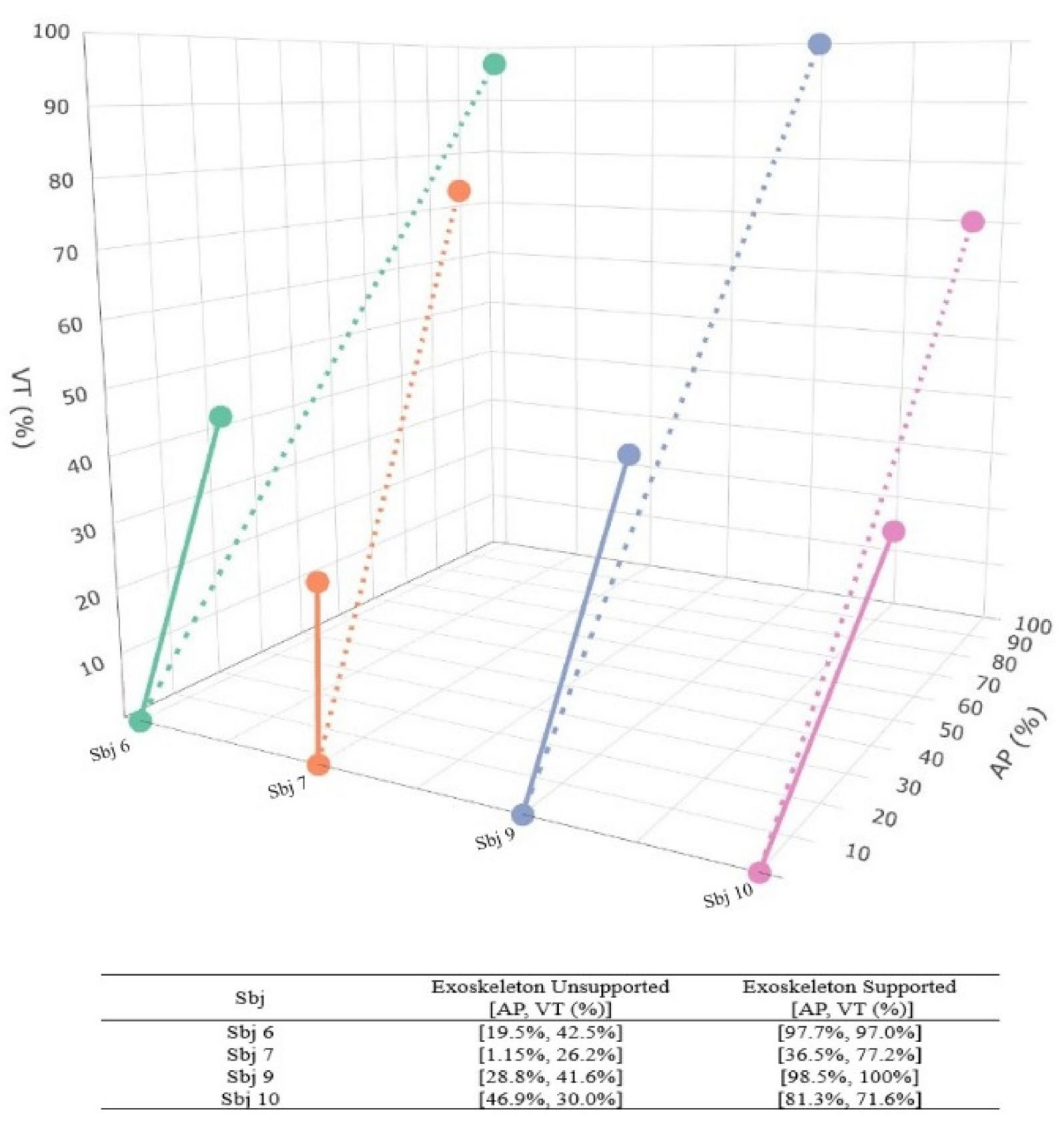
Median percentage of the upper limb vertical VT (%) and anteroposterior AP (%) travelled distance in the instrumented items: grasp a wood block with i) a 2.5 cm edge and ii) a 5 cm edge of the ARAT in the *Exoskeleton Unsupported* (solid line) and *Exoskeleton Supported* (dotted line) conditions. VT: Vertical, AP: Anteroposterior, Sbj: Subject, ARAT: Action Research Arm Test

Conversely, four participants (Sbj 6, Sbj 7, Sbj 9 and Sbj 10) were unable to complete the three instrumented items without assistance. Figure 9 shows the median percentage of the maximum values for both the vertical (VT) and anteroposterior (AP) directions across the two ARAT instrumented items selected for the kinematics analysis (i.e., grasping a wooden block with i) a 2.5 cm edge and ii) a 5 cm edge) under the two conditions: Exoskeleton Unsupported (solid line) and Exoskeleton Supported (dotted line). Specifically, in the *Exoskeleton Unsupported* condition, the four participants achieved 35.8% [29.0% – 41.9%] in the VT direction and 24.1% [14.9% – 33.3%] in the AP direction. In contrast, with Exoskeleton Support, their performance improved, reaching 87.1% [75.8% – 98.6%] in the VT direction and 89.5% [70.1% – 97.9%] in the AP direction.

**Fig. 10:**
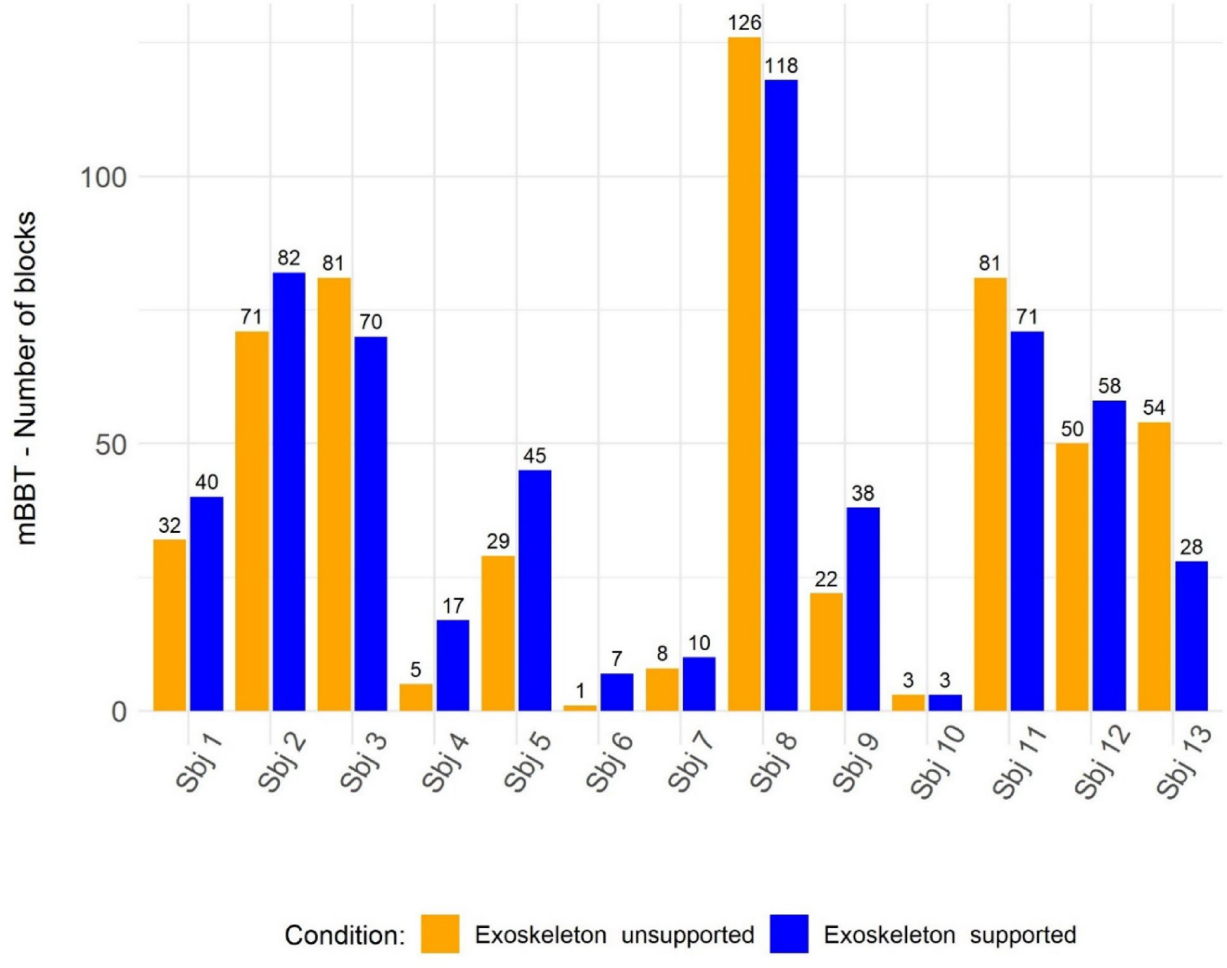
The orthotic effect of each participant performing the mBBT. Sbj: Subject; mBBT: modified Box and Block Test

The second part of the orthotic effect was evaluated based on the results in the mBBT (Fig. 10). Overall, the group showed limited positive effect of the exoskeleton support, with a median difference between *Exoskeleton Supported* and *Exoskeleton Unsupported* conditions of $\:{\Delta\:}mBBT=\:$6 [-8 – 11] transported blocks (W = 29.5, *p* = 0.46). Eight participants (Sbj 1, Sbj 2, Sbj 4, Sbj 5, Sbj 6, Sbj 7, Sbj 9, and Sbj 12) showed an improvement in the *Exoskeleton Supported* condition with a median of 39.1 [15.25–48.25] transported blocks compared to 25.5 [7.25–36.50] blocks in the *Exoskeleton Unsupported* condition. Four participants (Sbj 3, Sbj 8, Sbj 11 and Sbj 13) transported fewer blocks with exoskeleton support with a median of only 70.5 [59.5–82.75] transported blocks compared to a median of 81 [74.25–92.25] blocks in the *Exoskeleton* U*nsupported* condition (W= 11, p = 0.47). Sbj 10 showed no changes.

### Correlation analysis

The Spearman’s correlation coefficient between the $\:{\Delta\:}ARAT$ and the MMT_Shoulder Flexion is −0.71 (*p* = 0.006) while between $\:{\Delta\:}ARAT$ and the MMT_Shoulder Abduction is −0.59 (*p* = 0.032).

The Spearman’s correlation coefficient between the $\:{\Delta\:}ARAT$ and the MAS_Shoulder Flexion is 0.66 (*p* = 0.014), between the $\:{\Delta\:}ARAT$ and the MAS_Shoulder Abduction is 0.76 (*p* = 0.003) and between the $\:{\Delta\:}ARAT$ and the MAS_Elbow Extension is 0.59 (*p* = 0.034).

**Fig. 11:**
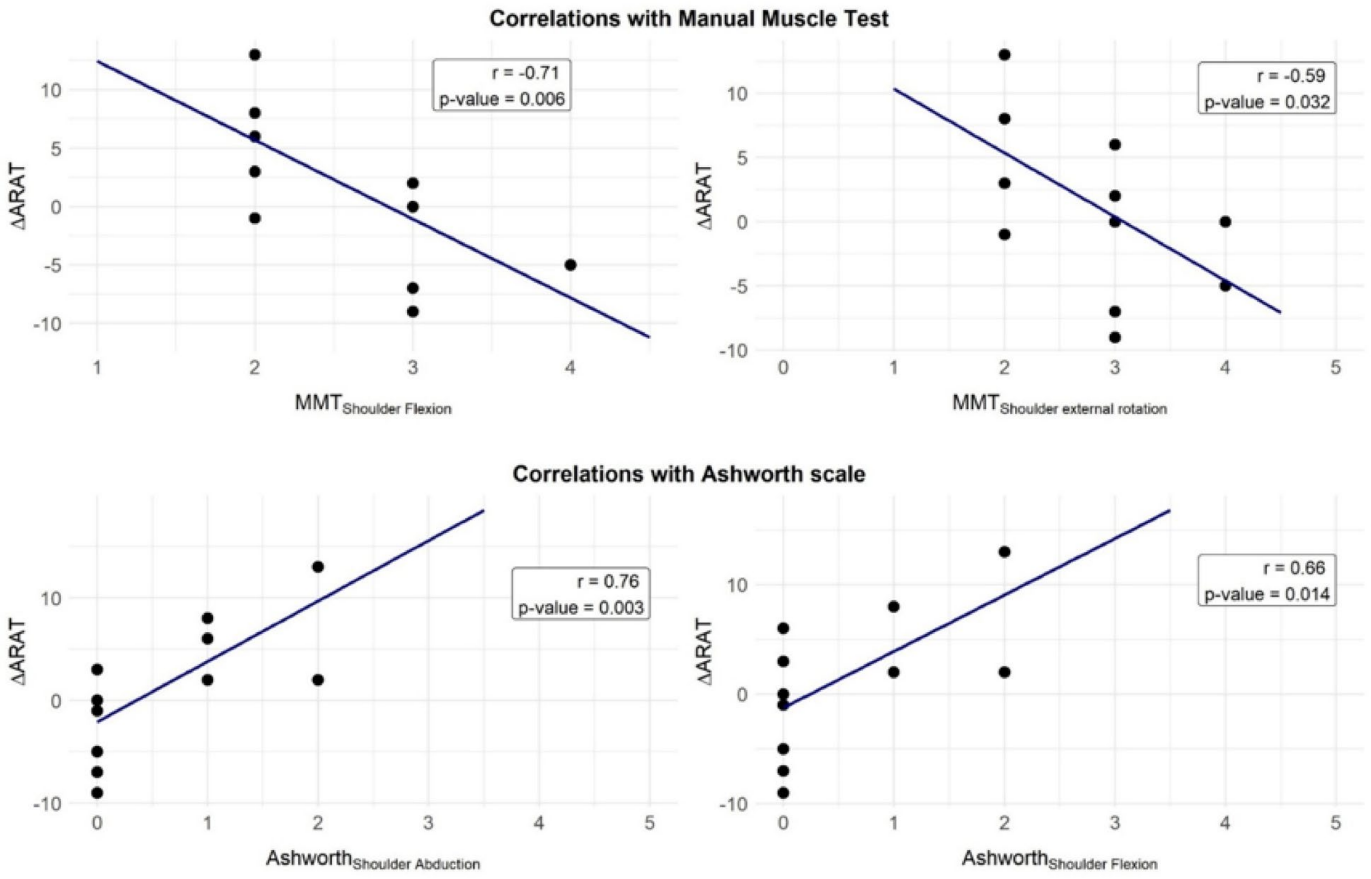
Spearman correlation analysis of the $\:\varvec{\Delta\:}$ARAT vs. shoulder flexion and abduction MMT and Ashworth

Finally, between the $\:{\Delta\:}ARAT$ and the ARAT_*Exoskeleton Unsupported* is −0.66 (*p* = 0.015) while between the $\:{\Delta\:}ARAT\:$ and the mBBT_*Exoskeleton Unsupported* is −0.57 (*p* = 0.042). All significant correlations are reported in figure 11. No significant correlations were found between the $\:{\Delta\:}mBBT$ and other clinical variables.

### Usability

All subjects completed the protocol with no adverse events. The SUS scores measured at the end of the experimental procedures were analyzed considering 68 as a threshold for acceptability and 80.3 as the threshold for a full positive evaluation [24]. The SUS median score of the whole group is 70 [62.5–70] points. Four participants (Sbj 1, Sbj 5, Sbj 7, Sbj 12) showed a score higher than 80 points, four participants (Sbj 2, Sbj 3, Sbj 6, Sbj 9) showed a score between 68 and 80 points and four participants (Sbj 4, Sbj 8, Sbj 10, Sbj 11) showed a score between 50 and 68 (one 67.5 near to be acceptable). Only Sbj 13 reported a score of 40 points as reported in Table 4.

**Table 4:** Usability assessment of the ASSISTarmMS using the SUS

Items	Sbj 1	Sbj 2	Sbj 3	Sbj 4	Sbj 5	Sbj 6	Sbj 7	Sbj 8	Sbj 9	Sbj 10	Sbj 11	Sbj 12	Sbj 13
I think that I would like to use this system frequently	5	5	4	4	5	5	4	3	3	3	4	4	2
I found the system unnecessarily complex.	1	3	2	3	3	2	2	3	2	2	3	2	3
I thought the system was easy to use.	5	4	4	3	5	4	4	3	4	4	3	4	2
I think that I would need the support of a technician to use the system.	4	4	3	5	3	4	1	4	4	4	5	1	5
I found the various functions in this system were well integrated.	4	4	4	3	5	4	4	3	4	4	3	4	3
I thought there was too much inconsistency in this system.	1	2	2	3	1	2	1	3	2	1	3	3	3
I imagine that most people would learn to use this system very quickly.	4	4	4	4	4	4	5	3	5	4	4	5	4
I found the system very cumbersome to use.	1	2	3	2	1	3	1	2	1	2	2	1	4
I felt very confident using the system.	5	3	3	4	5	3	5	3	4	3	4	5	3
I needed to learn a lot of things before I could get going with this system.	3	1	1	2	3	1	1	1	1	2	2	2	3
**SUS score**	**82.5**	**70**	**70**	**57.5**	**82.5**	**70**	**90**	**55**	**75**	**67.5**	**57.5**	**82.5**	**40**

*Sbj* Subject,* SUS* System Usability Scale

### Phase II – key findings

From a clinical standpoint, four subjects (Sbj 6, Sbj 7, Sbj 9, Sbj 10) exhibited severe upper-limb impairment, which prevented them from completing the three instrumented ARAT items without the exoskeleton. These subjects had significant limitations in muscle strength (Sbj 6 and Sbj 9) or showed relevant upper-limb spasticity (Sbj 7 and Sbj 10), restricting their ability to move against gravity. Their performance in the initial manual dexterity test (9HPT and BBT) was also poor, with fewer than thirteen blocks transported, well below the thirty-five-blocks threshold reported by Solaro et al. [27], confirming severe impairments in fine and gross motor skills. When using the exoskeleton, these subjects exhibited increased anteroposterior and vertical movement in the two instrumented items of the ARAT showing an improvement of 8 [6.5–9.25] points in their overall ARAT score. Additionally, subjects 6, 7, and 9 transported six, two, and sixteen more blocks, respectively, during the endurance test (mBBT). These improvements suggest a positive orthotic effect on functional capabilities for these severely impaired participants.

In contrast, nine subjects (Sbj 1, Sbj 2, Sbj 3, Sbj 4, Sbj 5, Sbj 8, Sbj 11, Sbj 12, Sbj 13) were able to complete the three instrumented ARAT items without the exoskeleton, demonstrating better movement performance. These subjects showed mild to moderate upper-limb impairment, as evidenced by muscle strength, spasticity, and functional test results (Table 3). For these individuals, the orthotic effect was negative or mixed. Specifically, subjects 8, 11 and 13 exhibited a negative orthotic effect as they needed 2.63 [2.17–3.45] seconds more to complete the three instrumented ARAT items increasing the Jerk Index by 0.53 [0.51–0.68], indicating reduced movement smoothness and motor control in the *Exoskeleton supported* condition [25]. Moreover, their overall ARAT decreased by 7 [6–8] points and they transported 10 [9–18] blocks less in the endurance test (mBBT). These subjects had upper-limb muscle strength ≥ 3, no spasticity, and adequate fine and gross motor skills.

Conversely, subjects 1, 2, 4, and 12 exhibited a mixed orthotic effect. Specifically, they needed 0.98 [0.61–1.11] seconds more to complete the three instrumented ARAT items increasing the Jerk Index values by 0.25 [0.21–0.25], with minimal or no improvement in the total ARAT score. However, they transported 9.5 [8-11.25] more blocks in the mBBT, suggesting a positive orthotic effect in terms of enhanced endurance. Given their mild upper-limb disability, it is possible that the device acted as a hindrance during the ARAT, without providing a significant advantage in the instrumental assessment or overall performance.

Notably, three of these subjects (Sbj 1, Sbj 2, and Sbj 12) reported fatigue levels exceeding the 50-point raw score cut-off on the FSS scale (equivalent to a corrected threshold of 5.5 points), indicating a severe fatigue burden [28]. This suggests that despite its limitations in functional tasks, the device could still be beneficial in endurance-related activities.

Subject 5 was the only one to show a six-point improvement in the ARAT, despite worsened performance in the three instrumented ARAT items. Additionally, he transported sixteen more blocks in the mBBT while using the ASSISTarmMS. This finding suggests that moderate upper-limb impairment, combined with fatigue, may represent a threshold beyond which the exoskeleton remains beneficial.

Finally, subject 3 showed a slight improvement in the overall ARAT score, but his performance in the endurance test deteriorated (he carried nine fewer blocks). His low level of fatigue may explain why the support provided by the device did not result in any long-term compensatory benefits.

Regarding usability, the group’s average score met the acceptability threshold defined by Bangor et al. [29]. However, perceptions varied significantly among participants. Subject 13 rated usability as ‘poor’ (unacceptable score on the SUS scale) while subjects 4, 8, 10, and 11 rated usability as ‘marginal’ (almost acceptable), with scores below 68 points. Except for subject 10, these participants did not experience a positive orthotic effect, which may explain their lower usability ratings. Although subject 10 demonstrated a “positive” orthotic effect, he rated the system as ‘almost acceptable’ likely because his improvements in the overall ARAT were not clinically significant. Furthermore, the ASSISTarmMS did not address his severe fine manipulation deficits which may have influenced his usability assessment.

The remaining eight subjects rated usability above the acceptability threshold: subjects 2, 3, 6 and 9 rated it as “good” while subjects 1, 5 and 12 rated it as “excellent”. Finally, subject 7 gave the highest usability rating (> 90 points, ‘best usability’). Subjects who experienced a clinically significant positive orthotic effect tended to rate the device more favourably. However, considering the prototype nature of the ASSISTarmMS and given that these evaluations were based on a single use, extreme values should be interpreted with caution.

## Discussion

The research consisted of two phases: Phase I identified design and usability issues by testing the original version of the ASSISTarmMS with three PwMS and two therapists, leading to a redesign of the original version of the exoskeleton; Phase II evaluated the orthotic effect and usability of the redesigned system in a group of thirteen users with MS to inform future exoskeleton design for this neurologically impaired population.

The redesigned exoskeleton, ASSISTarmMS, successfully addressed the previous limitations by enhancing safety, comfort, and usability. Key improvements included a quick-disconnect mechanism for increased safety, customizable shells and padding for better comfort, simplified donning and doffing procedures, and enhanced adjustability. These refinements are crucial, as traditional exoskeletons often suffer from rigid structures and inadequate adaptability, leading to discomfort, poor compliance, and limited practicality in rehabilitation settings [30]. Moreover, complex donning and doffing processes discourage regular use, particularly among individuals with neurological impairments. Safety concerns, such as the absence of quick-release mechanisms, further hindered their adoption [31]. The redesigned ASSISTarmMS seems to be aligned with the principles outlined by Gull et al., emphasizing the importance of multidisciplinary collaboration and user-centred design in assistive technology development [31]. By emphasizing safety, comfort, and usability, ASSISTarmMS addressed some of the key challenges associated with the adoption of exoskeletons in rehabilitation, potentially supporting improved outcomes and broader clinical applications. Additionally, Phase I of the study helped establish a methodological framework for iterative device refinement, integrating clinical feedback with engineering adjustments. These insights contributed to enhancing the immediate functionality of ASSISTarmMS, facilitating Phase II of the study, and offering a useful reference for future exoskeleton development.

Phase II of the study revealed both a positive and a negative orthotic effect. The positive one was measured as an increment of functional capacity or endurance in the primary outcomes. Conversely, the negative one was mainly assessed as a decline in movement performance during instrumental assessment, which resulted in either a deterioration or no change in the primary outcome measures.

The orthotic effect was positive on PwMS with moderate to severe upper-limb impairment, especially considering muscle strength and spasticity. This conclusion is bolstered by the correlation analyses, which demonstrated a significant inverse relationship between participants’ change score in the primary outcome, measuring upper-limb function, and muscle strength, alongside a significant direct relationship with the level of upper-limb spasticity. It is important to notice that participants who did not show severe spasticity (≥ 3) hindering movement and maintained fine motor skills benefited the most from the device.

Considering the negative orthotic effect, this was mainly observed in participants with mild upper-limb impairment, suggesting that the device may hinder rather than enhance movement performance for PwMS capable of performing 3D reaching movements against gravity. This could be likely due to the inertia of the counterbalancing weight at physiological movement velocities, which may require significant effort from the user to decelerate the movement at the end of the forward phase and reinitiate it in the backward phase, making movement execution more challenging. However, despite a decline in movement smoothness, subjects with high fatigue levels showed improvements in the endurance test, suggesting that the exoskeleton may provide an advantage in muscle endurance rather than fine motor function.

The usability assessment generally confirms these findings, with participants with moderate to severe impairments giving positive usability assessment compared to those with less severe upper-limb impairments, who provided lower scores and primarily reported potential discomfort.

Compared to our previous study with the original exoskeleton [22], the redesigned ASSISTarmMS showed improved performance and usability. In the earlier study, the most impaired participants gained + 3.3/15 points on a simplified ARAT and increased wrist displacement by 22%, but usability was low (SUS scores: 30 and 52). In contrast, in the present study, severely impaired PwMS improved by + 8 points on the full ARAT, with greater wrist displacement (+ 35.8% vertical; +24.1% anteroposterior) and a median SUS score of 70 for usability. These findings support the added clinical value of the redesigned system.

Based on the observed outcomes, the ASSISTarmMS may be suitable for future rehabilitative applications. These include gravity-compensated functional movement and upper-limb endurance training. In individuals with proximal weakness or mild spasticity, the device can assist shoulder elevation and elbow extension against gravity. This support may enable reaching or object-moving exercises at various heights. The exoskeleton could also serve as a preparatory or assistive tool during standard rehabilitation of upper-limb activities of daily living, such as object manipulation or personal care simulations, that are typically unfeasible due to upper-limb impairments. Moreover, the device could facilitate repetitive gross motor tasks aimed at enhancing upper-limb endurance in individuals with higher fatigue severity.

### Limitations and future developments

This study highlights several limitations related to the study methodology and the design of the ASSISTarmMS exoskeleton. A major limitation, even after the redesign phase of this study, remains the device bulkiness. In particular, the central stand restricts portability, and confines use to clinical settings limiting its potential impact on daily life. Future iterations will prioritize a more compact, lightweight design (e.g., such as modular configurations or direct wheelchair mounting) to enable home-based rehabilitation and community settings. While the exoskeleton bilateral compatibility was useful in research settings, it proved impractical for routine clinical use. A more streamlined approach, with dedicated right and left-arm versions, could improve usability and reduce unnecessary bulk. Additionally, lighter materials like carbon fiber and aluminium could be used to further reduce the device weight.

Another challenge is the counterweight system balancing the user’s arm weight. Future designs should replace it with an adjustable spring mechanism to streamline setup, minimize inertia effects, and improve efficiency. This modification could potentially enhance arm support, responsiveness, and adaptability for various tasks.

Moreover, the exoskeleton lacks hand-function support. While effective for shoulder and elbow movements, it does not aid grasping, limiting usability for MS subjects with severe manipulation impairment. Integrating Functional Electrical Stimulation (FES) with the ASSISTarmMS could enhance grasp and release functions, making the system more effective for users with significant motor deficits.

Considering the study methodology, the small sample size limits the generalizability of findings, reducing statistical power. Larger-scale studies are needed to validate these preliminary results and ensure broader applicability for PwMS. Finally, phase II focused solely on short-term orthotic effects, leaving long-term benefits unexplored. Future research should incorporate longitudinal studies to assess the sustained impact on motor function, fatigue management, and daily independence. Such studies would clarify whether the ASSISTarmMS, or passive arm support in general, contribute to meaningful rehabilitation improvements over time in PwMS. Addressing these limitations through design refinements, expanded testing, and robust longitudinal research will be crucial for optimizing the ASSISTarmMS exoskeleton.

## Conclusion

This study highlights the importance of balancing assistive functionality with usability and user experience. Phase I underscores the importance of an iterative collaboration between therapists and engineers in the redesign process of the ASSISTarmMS.

Phase II provides evidence supporting the potential of the ASSISTarmMS exoskeleton as a passive gravity-compensated assistive device for PwMS. The findings demonstrate that the exoskeleton can enhance upper-limb function and endurance in individuals with moderate to severe impairments, particularly those experiencing muscle weakness or spasticity. Indeed, for PwMS with mild motor deficits, the device may alter movement dynamics, affecting natural execution.

Further mechanical enhancements are essential to optimize the exoskeleton usability in rehabilitation settings. Future refinements should focus on reducing bulk, streamlining setup procedures, and enhancing adaptability to better accommodate the diverse needs of users and clinical environments.

## Supplementary Information

Supplementary material 1

## Data Availability

The data supporting the conclusions of this article are included in the article and its supplementary materials.

## Abbreviations

9HPT: Nine Hole Peg Test
AP: Anteroposterior
ARAT: Action Research Arm Test
BBT: Box and Block Test
EDSS: Expanded Disability Status Scale
FES: Functional Electrical Stimulation
FSS: Fatigue Severity Scale
IMU: Inertial Measurement Unit
LIGHTarm: Name of the original exoskeleton
MAS: Modified Ashworth Scale
MMSE: Mini-Mental State Examination
MMT: Medical Research Council Manual Muscle Testing Scale
MS: Multiple Sclerosis
mBBT: Modified Box and Block Test
PwMS: People with Multiple Sclerosis
SBJ: Subject
SD: Standard Deviation
SUS: System Usability Scale
VT: Vertical
