# Supplementary material for: Investigating the orthotic effect of a passive gravity-compensated exoskeletal device on upper-limb function in people with multiple sclerosis: a pilot cross-sectional study
Source: J Neuroeng Rehabil. 2025 Aug 26;22:186. doi: 10.1186/s12984-025-01715-8 (PMC12382219; doi:10.1186/s12984-025-01715-8)
Supplement: Supplementary file 1 — Supplementary material 1 [file 12984_2025_1715_MOESM1_ESM.docx]

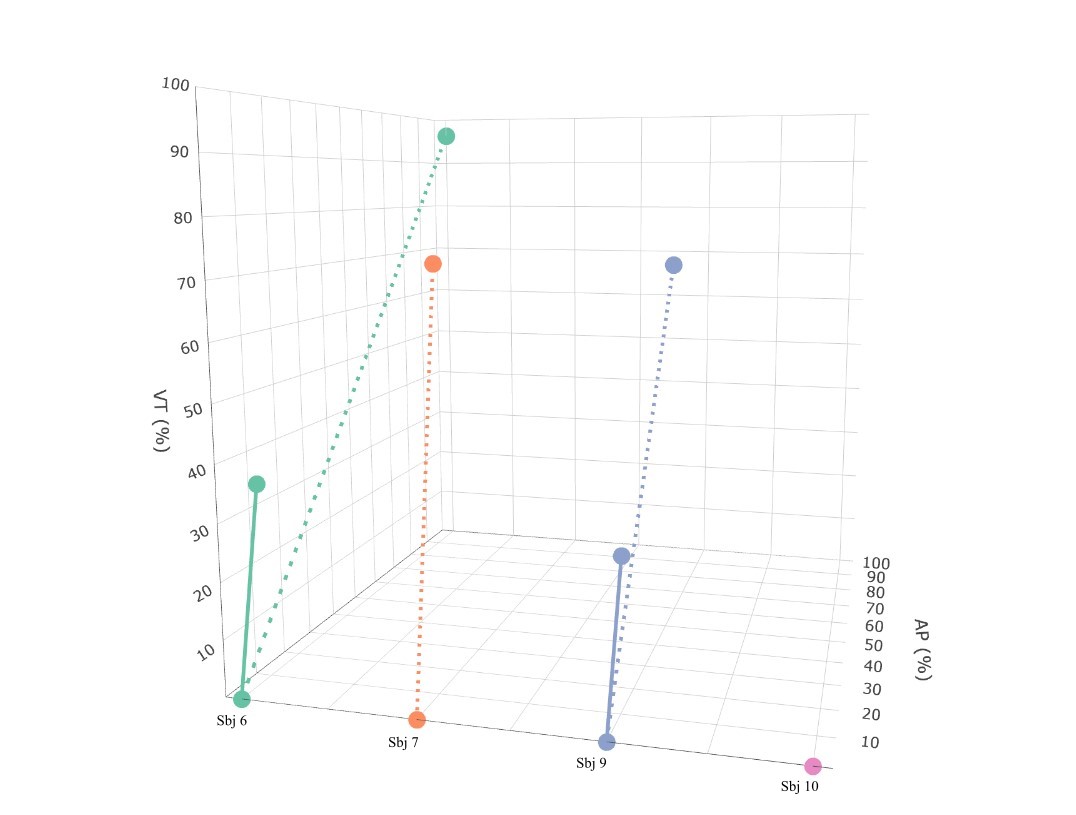


**Supplementary Figure 1. Median percentage of the upper limb vertical VT (%) and anteroposterior AP (%) travelled distance in the instrumented item “pinch a ball bearing with 2nd finger and thumb” of the ARAT in the Exoskeleton Unsupported (solid line) and Exoskeleton Supported (dotted line) conditions. VT: Vertical, AP: Anteroposterior, Sbj: Subject, ARAT: Action Research Arm Test.**
